# Extremely low frequency electromagnetic field exposure and restraint stress induce changes on the brain lipid profile of Wistar rats

**DOI:** 10.1186/s12868-018-0432-1

**Published:** 2018-05-21

**Authors:** Jesús Martínez-Sámano, Alan Flores-Poblano, Leticia Verdugo-Díaz, Marco Antonio Juárez-Oropeza, Patricia V. Torres-Durán

**Affiliations:** 1 Departamento de Bioquímica, Facultad de Medicina, Universidad Nacional Autónoma de México, Circuito Escolar s/n, Ciudad Universitaria, C.P. 04510 Mexico City, Mexico; 2 Departamento de Fisiología, Facultad de Medicina, Universidad Nacional Autónoma de México, Circuito Escolar s/n, Ciudad Universitaria, C.P. 04510 Mexico City, Mexico

**Keywords:** ELF-EMF, Corticosterone, Cholesterol, Fatty acids

## Abstract

**Background:**

Exposure to electromagnetic fields can affect human health, damaging tissues and cell homeostasis. Stress modulates neuronal responses and composition of brain lipids. The aim of this study was to evaluate the effects of chronic extremely low frequency electromagnetic field (ELF-EMF) exposure, restraint stress (RS) or both (RS + ELF-EMF) on lipid profile and lipid peroxidation in Wistar rat brain.

**Methods:**

Twenty-four young male Wistar rats were allocated into four groups: control, RS, ELF-EMF exposure, and RS + ELF-EMF for 21 days. After treatment, rats were euthanized, the blood was obtained for quantitate plasma corticosterone concentration and their brains were dissected in cortex, cerebellum and subcortical structures for cholesterol, triacylglycerols, total free fatty acids, and thiobarbituric acid reactive substances (TBARS) analysis. In addition, fatty acid methyl esters (FAMEs) were identified by gas chromatography.

**Results:**

Increased values of plasma corticosterone were found in RS and ELF-EMF exposed groups (p < 0.05), this effect was higher in RS + ELF-EMF group (p < 0.05, vs. control group). Chronic ELF-EMF exposure increased total lipids in cerebellum, and total cholesterol in cortex, but decreased polar lipids in cortex. In subcortical structures, increased concentrations of non-esterified fatty acids were observed in RS + ELF-EMF group. FAMEs analysis revealed a decrease of polyunsaturated fatty acids of cerebellum and increases of subcortical structures in the ELF-EMF exposed rats. TBARS concentration in lipids was increased in all treated groups compared to control group, particularly in cortex and cerebellum regions.

**Conclusions:**

These findings suggest that chronic exposure to ELF-EMF is similar to physiological stress, and induce changes on brain lipid profile.

## Background

Brain lipids have different roles such as structural, functional and metabolic. Brain lipids constitute about one-half of brain-tissue dry weight. The brain has fatty acids that are long-chain monocarboxylic acids, either saturated or unsaturated, and cholesterol; the most prevalent families of unsaturated fatty acids are n-3, n-6, and n-9 series and contains some unusual fatty acids, such as very long chain fatty acids [1]. However, fatty acids are associated to other compounds to form glycerophospholipids and sphingolipids. These lipids play important roles in the brain physiology, e.g. in signal transduction across membranes, formation of lipid rafts, and anchoring the cell membranes to the extracellular matrix; furthermore, lipids covalently coupled to proteins play a major role in anchoring marker proteins within membranes [2].

Cholesterol and phospholipids are the main constituents of biomembranes. Changes in membrane lipid content are usually associated to imbalance in lipid homeostasis. In central nervous system (CNS) the lipid imbalance could lead to functional alterations that could result in several pathologies [1] as Alzheimer, Huntington and Parkinson diseases [3].

Stress could be defined as a condition in which homeostasis is modified by several responses, physiological and adaptive, induced by stressing factors. Hypothalamic–pituitary–adrenal (HPA) axis and the autonomic nervous system enhance the stress hormones in plasma, glucocorticoids and catecholamines, respectively [4, 5]. Stress exposure also induces deep changes in brain functions. Chronic stress has been related to increase in oxidative parameters for instance, increase in protein and lipid peroxidation, increased activity of antioxidant enzymes in the cortex, hippocampus and cerebellum [6]. However, the stress effects on brain lipids have not been extensively characterized.

Nowadays, the presence of electromagnetic fields in daily life it has resulted in an increase concerns regarding to the potential adverse effects of exposure to non-ionizing radiation; particularly to extremely low frequency electromagnetic fields (ELF-EMF). The effects induced by ELF-EMF exposure on biological systems are unclear. However, some effects are reported in epidemiological studies due to the incidence of certain types of brain cancer, and mood disorders [7]. Recent studies have reported the possible oxidant effect of ELF-EMF in brain, through the actions of reactive oxygen species [8]. Some authors have considered ELF-EMF as a mild stressor, and the effects related to its exposure have been reviewed [9]. In a previous study, carried out in Wistar rats acutely exposed to ELF-EMF, we have shown that they increase serum non-esterified fatty acids (NEFAs) concentration at 24 h post exposure [10] and impairs the antioxidant status of rat brain [11]. There are reports that show changes on the levels of Thiobarbituric Acid Reactive Substances (TBARS) of rats exposed to ELF-EMF in the cerebral cortex [12] or in various brain areas [13]. However, the effects of ELF-EMF on brain lipids and their metabolism have not been elucidated extensively. The aim of the present study was to evaluate the effects of chronic extremely low frequency electromagnetic field (ELF-EMF) exposure, restraint stress (RS), and both (RS + ELF-EMF) on lipid profile and lipoperoxidation status in cortex, cerebellum, and subcortical structures of Wistar rat brain.

## Methods

### Reagents

All reagents and chemicals used for the buffers and solution preparation were of analytical grade. Chloroform and methanol were purchased from Merck (Mexico City, Mexico). Kits for the assessment of Total cholesterol (TC) and triacylglycerols (TAG) were purchased from Spinreact (Mexico City, Mexico), and kits for Non Esterified Fatty Acids from Roche (Mexico City, Mexico).

### Animals and treatments

Male Wistar rats (aged 8 weeks) were bred in Faculty of Medicine, UNAM. A total of 24 young rats were used in the experiments (weight 180–200 g); the rats were acclimated to the room and light conditions by 3 days before the experimental period. The animals had free access to water and food in a room with controlled temperature of 23 °C ± 2 and 12 h light–dark cycles. The animals were placed in acrylic homecages of 47 × 21 × 25 cm. All procedures involving animal care were conducted in compliance with the guidelines of animal care, and previously approved by the ethics and research committee of UNAM School of Medicine. All efforts were made to minimize the number of animals used and their suffering. Rats were randomly assigned to four groups of 6 rats each: Control (C), intact animals (nonstressed, 21 days); Restraint stress (RS), (positive control for stress, 2 h/day for 21 days); Extremely low frequency electromagnetic field exposure (ELF-EMF, unrestrained, 2 h/day for 21 days); Restraint stress plus ELF-EMF exposure (RS + ELF-EMF, 2 h/day for 21 days).

After 21 days of treatment and immediately after last exposure, rats were euthanized by cervical dislocation and their blood was collected into heparinized tubes. Right away the brain was dissected in cortex, cerebellum and subcortical structures and stored at − 70 °C until analysis.

### Restraint stress model (RS)

The model of movement restraint was used as a positive control for physical and psychological stress [14]. Movement restraint was performed by placing the animals into acrylic cylinders (18 cm in length × 7 cm in diameter) for 120 min/day during 21 days from 12:00 to 14:00 h. Unrestrained rats were individually placed in acrylic homecages during the same period into the same experimental room.

### Extremely low frequency electromagnetic field exposure

ELF-EMF exposure was applied with a device previously used in our laboratory [15]. The electromagnetic field was generated with a pair of Helmholtz coils (30 cm internal diameter) composed of 18 gauge copper wire in 350 turns. When electrical current pass through the coils in the same direction, it creates a highly uniform magnetic field in a 3-dimension region of space inside the coils. Helmholtz coil is a device for producing a region of nearly uniform magnetic field. The coils were connected in parallel to a 120 V adjustable transformer (Staco Energy Products, Dayton, OH). An oscilloscope (Tecktronix 5103N, Beaverton, OR) was coupled to the system to monitor a 60 Hz sinusoidal magnetic waveform. The amplitude of the magnetic flux density was 2.4 mT, which was measured using a hand-held Gauss/Tesla meter (Alpha Lab, Salt Lake City, UT). Helmholtz coils were in an isolated room containing also the control animals out of the electromagnetic exposure area. Background static magnetic field value was about < 0.4 µT in the room where all animals were kept and in the central area of the switched off coils. The temperature inside the exposure chambers was 23.4 ± 0.4 °C. The temperature between the coils was monitored using a Hygro-thermometer (Extech instruments, Waltham, MA), this parameter remained constant during the 2 h of stimulation. The coils were separated 15 cm from the upper and lower surfaces of the animal cage [11, 15]. ELF-EMF exposure was applied during the same time (from 12:00 to 14:00 h) to the corresponding groups [15].

### Plasma corticosterone determination

Plasma corticosterone concentration was quantitated using an ELISA kit (Enzo Life Sciences, Farmingdale, NY, USA), and microplate reader Stat Fax 3200 (Awareness Technology Inc. Palm City, FL, USA) according to the manufacturer´s instructions.

### Brain lipid analysis

Total lipids of cortex, cerebellum and subcortical structures were extracted [16] with a chloroform/methanol mixture by a modified Folch’s method and gravimetrically evaluated: for cortex, cerebellum and subcortical structures samples, 0.5 g of fresh tissue was homogenized in 4 volumes of 0.05 M phosphate buffer, pH 7.2, containing 0.025% butylated hydroxytoluene as antioxidant. Then, the pH was adjusted to 6.0 by the addition of HCl dissolution, and this suspension was extracted three times with 3 volumes each of the chloroform/methanol mixture (Folch´s dissolvent). The extract was washed with 10 mL of water, the organic fraction was evaporated under a nitrogen stream, then weighed (for total lipids), and stored at − 70 °C prior to TC, TAG, NEFAs, and gas chromatography (GC) of fatty acid methyl esters (FAMEs) analyses were performed [17, 18]. The polar lipids (POL) were calculated as follows: POL = TL − (TC + TAG + NEFAs). All results were adjusted per mg total lipids (TL) and for measuring the absorbance it was performed with Genesis 10 UV spectrophotometer (Thermo Electron Corporation, Louisville, KY, USA).

### Gas chromatography–mass spectrometry analysis

FAMEs from brain lipid extract were analyzed according to Torres-Durán [18] by GC in a Hewlett Packard gas chromatograph (HP 5890; Hewlett Packard, Mexico City) coupled to a mass selective detector (HP model 5972), with helium as gas carrier; temperature program starts at 200 °C for 3 min, then increased to 260 °C at a ramp velocity of 4 °C/min during 15 min and then maintained at 260 °C for 7 min. Spectra were obtained at 70 eV ionizing energy and mass range scanned was 50–700 a.m.u. at 1.5 scan/s. Fatty acid methyl esters were identified by comparing retention times of authentic fatty acids (Supelco) and by their mass spectra. Column, fused silica capillary column (25 m × 0.2 mm, 0.2 mm i.d.) coated with dimethylpolysiloxane (0.33 μm film) Hewlett Packard, Ultra 1 HP.

### Lipoperoxides determination

Thiobarbituric acid reactive substances were determined according to Torres-Duran et al. [18]. Absorbance of the samples was interpolated in curves for concentration of malondialdehyde (MDA, ng/mg TL).

### Statistical analyses

Frequency distribution for variables was determined by Kolgomorov–Smirnov test. Comparisons between groups were done using ANOVA (corticosterone, MDA, total cholesterol and, polar lipids) using Bonferroni post hoc test for contrasts among groups or Kruskal–Wallis test (NEFAs and saturated/unsaturated fatty acid ratio).

## Results

### Effects of RS and ELF-EMF exposure on plasma corticosterone

**Fig. 1:**
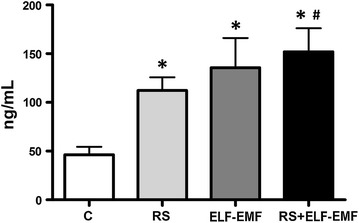
Effects of EMF chronic exposure and RS on plasma corticosterone. Results are expressed as mean ± SD of 6 animals in each group (*p < 0.05 vs. C group and ^#^p < 0.05 vs. RS group)

In order to visualize the effect of ELF-EMF on the stress status, we quantified the plasma corticosterone concentration. Increased values of plasma corticosterone were found in all the experimental groups (p < 0.05), this effect was higher in RS + ELF-EMF group compared to control group (C vs. RS + ELF-EMF and RS vs. RS + ELF-EMF, p < 0.05) (Fig. 1).

### RS and ELF-EMF effects on TL, TC, TAG, and POL

Total lipids concentration in all the experimental groups shows a tendency to decrease in cortex and subcortical structures (Table 1), the lowest value was observed in subcortical structures of the RS + ELF-EMF group. However, TL in the cerebellum shows an increase only in ELF-EMF group that was statistically significant.

**Table 1:** Effects of ELF-EMF chronic exposure and RS on total lipids, total cholesterol, polar lipids and NEFAS in brain regions

Parameter	Group	Brain region		
		Cortex	Cerebellum	Subcortical structures
Total lipids (TL, mg/g wet weight)	C	60.5 ± 9.6	59.6 ± 3.7	70.5 ± 10.0
	RS	46.6 ± 8.7**	47.6 ± 5.7	47.4 ± 5.9*
	ELF-EMF	53.4 ± 7.3	71.4 ± 10.3^&^	59.3 ± 4.7*
	RS + ELF-EMF	42.3 ± 7.5**	55.6 ± 10.2	33.6 ± 5.6^$,#^
Total cholesterol (TC, μg/mg TL)	C	159.7 ± 1.0	188.3 ± 27.5	99.0 ± 18.6
	RS	169.6 ± 9.7	182.0 ± 25.4	101.8 ± 13.3
	ELF-EMF	194.3 ± 10.6*^,&^	148.5 ± 42.7	144.3 ± 35.0
	RS + ELF-EMF	198.6 ± 24.8*^,&^	173.0 ± 34.4	194.3 ± 67.6*^,&^
Polar lipids (POL, μg/mg TL)	C	753.5 ± 10.4	763.6 ± 73.6	831.2 ± 23.7
	RS	745.2 ± 14.0	714.0 ± 39.4	839.5 ± 14.2
	ELF-EMF	716.5 ± 22.5*	771.7 ± 56.6	786.9 ± 50.8
	RS + ELF-EMF	720.5 ± 26.0*	731.7 ± 48.2	729.4 ± 74.5*,^&^
NEFAs (μg/mg TL)	C	10.6 ± 1.9	16.5 ± 7.6	7.6 ± 2.2
	RS	9.3 ± 1.3	25.8 ± 9.4	9.5 ± 3.2
	ELF-EMF	9.9 ± 2.7	15.1 ± 3.8	10.4 ± 2.5
	RS + ELF-EMF	9.4 ± 3.5	15.0 ± 6.3	15.7 ± 4.3*^,#^

Results are expressed as mean ± SD of 6 animals in each group. For total lipids: **p < 0.01 versus control group, *p < 0.05 versus RS and RS + EMF groups, ^$^p < 0.05 versus C group, and ^#^p < 0.05 versus RS and EMF groups; for total cholesterol and polar lipids: *p < 0.05 versus control group and ^&^p < 0.05 versus RS group; and NEFAS: *p < 0.01 versus control group and, ^#^p < 0.05 versus RS and EMF groups

Regarding with TC, the same table shows a significant increase on TC content in the cortex of ELF-EMF and RS + ELF-EMF groups, and in subcortical structures of RS + ELF-EMF group (p < 0.05), but without changes in the cerebellum. A decrease on POL content was found only in the cortex of ELF-EMF, and RS + ELF-EMF groups (p < 0.05 vs. C group). Subcortical structures in RS + ELF-EMF group showed lower POL values than in control and RS groups (p < 0.05). No statistical differences were found in cerebellum.

Triacylglycerol concentration was not different neither in the analyzed brain tissue (cortex, cerebellum, subcortical structures), nor in the four groups (C, RS, ELF-EMF, and RS + ELF-EMF). Their values were, in the range of 49.22–79.24 μg/mg TL.

The NEFAs content in cortex, cerebellum and subcortical structures are shown on Table 1. No statistically significant differences were found between groups and cerebral regions analysed, except in subcortical structures, where the exposure to RS + ELF-EMF induced a slight NEFAs increase compared to the RS and C groups, causing 1.6 and 2.0-fold increases, respectively (p < 0.05).

### Effects of RS and ELF-EMF exposure on FAMEs relative content in brain regions

**Fig. 2:**
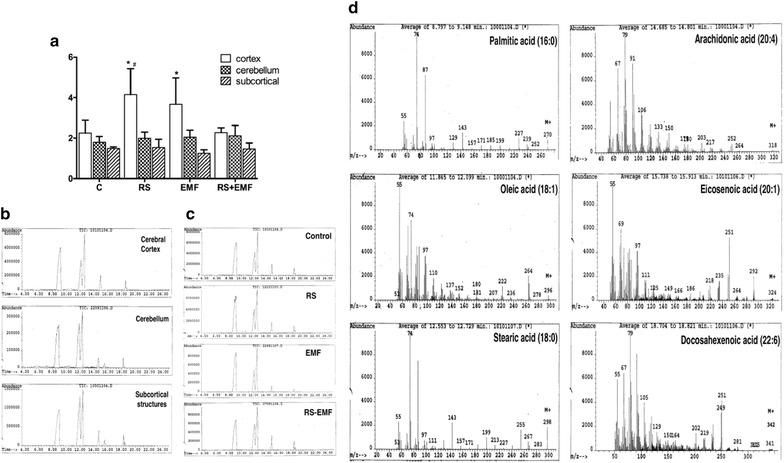
Effects of EMF chronic exposure and RS on FAMEs in brain regions. **a** FAMEs saturated/unsaturated ratio. Results are expressed as mean ± SD of 6 animals in each group (*p < 0.05 vs. control group and ^#^p < 0.05 vs. RS + EMF group). **b** Representative total ion chromatogram from each isolated region of rat brain from control group. Fatty acids are shown in order of appearing: palmitic (16:0), oleic (18:1), stearic (18:0), arachidonic (20:4) eicosenoic (20:1), docosahexenoic (22:6) and adrenic (22:4). **c** Representative total ion chromatogram of fatty acids lipid profile from cerebral cortex on different treatments. **d** Representative mass spectra of fatty acids obtained by relation mass/charge. Each fatty acid was identified by its specific molecular ion, base peak, and characteristic ions. Palmitic (16:0), oleic (18:1), stearic (18:0), arachidonic (20:4), eicosenoic (20:1), docosahexenoic (22:6), methyl ester

The FAMEs profile showed that palmitic acid was the most abundant, with a range 33–51% in relative abundance in all analysed tissues (data not shown). For stearic acid, a 23–28% relative abundance was found in cortex and cerebellum and 32–36% in subcortical structures. In addition, the percentage of total unsaturated fatty acids was 15–40% in the studied brain regions. Stress induced by RS or ELF-EMF caused an increase in the saturated/unsaturated fatty acid ratio in the cortex (p < 0.05 vs. control group), but not in other analyzed brain regions (Fig. 2). This increase was due to palmitic acid in RS group (51 ± 8.5 vs. 39.4 ± 4.4, p < 0.05 vs. C group). Finally, a significant increase of stearic acid was found in the cerebellum when the rats were exposed to ELF-EMF (27.4 ± 2.0 vs. 23.5 ± 1.9 p < 0.05 vs. control group), but not in other groups (data not shown).

The following unsaturated fatty acids were identified in the brain regions studied: eicosatetraenoic, docosahexaenoic, docosatetraenoic, octadecenoic and eicosenoic acids; however, only three unsaturated fatty acids (20:4, 22:4, and 22:6) showed differences among the treatments (Table 2). In brain cortex RS induced a decrease of eicosatetraenoic and docosahexaenoic acids percentages (p < 0.05 vs. C group), a decrease in docosatetraenoic acid was found in ELF-EMF group compared to C group (p < 0.05). In the cerebellum, we found a decrease of eicosatetraenoic and docosahexaenoic acids in ELF-EMF, and RS + ELF-EMF groups (p < 0.05 vs. C group). Cerebellum’s docosatetraenoic acid was only detected in control group. Finally, in subcortical structures it was found an increase of all unsaturated fatty acids in ELF-EMF group (p < 0.05) when compared to control group, and to other treatments.

**Table 2:** Effects of ELF-EMF and RS exposure on FAMEs in brain regions

Relative abundance	% Eicosatetraenoic (20:4)	% Docosahexaenoic (22:6)	% Docosatetraenoic (22:4)
Cortex			
C	4.9 ± 1.2	2.2 ± 1.1	0.8 ± 0.4
RS	1.9 ± 1.0*	0.7 ± 0.1*	0.5 ± 0.3
ELF-EMF	2.7 ± 1.4*	0.8 ± 0.5*	0.3 ± 0.2*
RS + ELF-EMF	5.4 ± 0.8^#^	2.8 ± 0.8^#^	0.7 ± 0.2
Cerebellum			
C	2.8 ± 0.8	1.5 ± 0.8	0.3 ± 0.2
RS	1.9 ± 0.7	0.8 ± 0.5	ND
ELF-EMF	1.3 ± 0.6*	0.6 ± 0.4*	ND
RS + ELF-EMF	1.3 ± 0.5*	0.3 ± 0.2*	ND
Subcortical			
C	4.8 ± 0.7	2.5 ± 0.5	0.8 ± 0.2
RS	3.1 ± 1.5^&^	1.5 ± 0.9^&^	0.4 ± 0.3^&^
ELF-EMF	6.5 ± 0.6*	4.1 ± 1.3*	1.5 ± 0.5*
RS + ELF-EMF	4.4 ± 1.0^&^	2.4 ± 0.8^&^	0.6 ± 0.3^&^

Octadecenoic (18:1) and eicosaenoic (20:1) acids were found in all regions, average 20–30 and 0.8–2%, respectively, without differences versus control group. Results are expressed as mean ± SD of 6 animals in each group (*p < 0.05 vs. control group, ^&^p < 0.05 vs. ELF-EMF group, and ^#^p < 0.05 vs. RS and EMF groups)

*ND* no detected

### Effects of RS and ELF-EMF exposure on TBARS concentration in lipids of brain regions

**Fig. 3:**
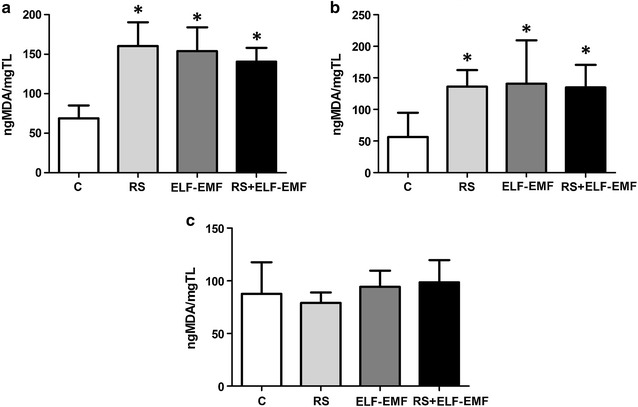
Effect of EMF chronic exposure and RS on the end products of oxidation (TBARS). Results are expressed as mean ± SD of 6 animals in each group. **a** MDA in cortex (*p < 0.05 vs. C group). **b** MDA in cerebellum (*p < 0.05 vs. C group). **C** MDA in subcortical structures

TBARS (MDA concentration) in lipids was increased in all the treated groups compared to control group (p < 0.05), in cortical and cerebellar regions (Fig. 3). No statistical differences were found among the groups in subcortical structures.

## Discussion

The magnetic flux density used in this work was half the limit recommended for occupational exposures to 50/60 Hz magnetic fields, which is 5 mT for short term exposure (maximum exposure duration is 2 h per workday) [19]. Our results demonstrate that ELF-EMF exposure induces a significant increase in plasma corticosterone levels, indicating a chronic stress status similar to induced by RS. Although major stress was found in RS + ELF + EMF group, there isn’t statistical difference between ELF + EMF and RS + ELF + EMF treatments. Nevertheless, this finding suggests a possible interaction, synergic or additive, that deserves further studies.

The RS model can induce deep changes in rat physiology; Hennebelle found an increment of 30-times basal corticosterone concentration when RS was applied 6 h/day for 21 days [20]. Buynitsky and Mostofsky [21] reported that a daily 3-week period of RS is used in rodents like a chronic stress physiological model, with an increase of corticosterone levels and metabolic perturbations that affects the function of the nervous system. In the present study, the three times increased corticosterone concentration on the exposure protocol supports the statement that RS and ELF-EMF alone, can act as a mild stress condition. These results are in accordance with previous studies, showing that plasma corticosterone levels are one of the most important indicators of stress [22, 23]. Some reports suggest that long-term ELF-EMF exposure may elevate the plasma corticosterone levels in rodents [23]. Taken together, RS + ELF-EMF may count as the addition of two stress situations, whose effects can be added. In the present study, an increase on corticosterone levels was found in RS, ELF-EMF, and RS + ELF-EMF groups, supporting the proposal that ELF-EMF exposure is like a mild stressor.

Lipids play a critical role in structure and function of the nervous system; glucocorticoids may alter lipid metabolism in brain and other tissues; as reported previously, where the administration of glucocorticoids can induce a shift in arachidonic acid metabolism in brain [24]. In the present study, we found changes on TC (increase), and POL (decrease) in the brain cortex of rats exposed to RS + ELF-EMF and ELF-EMF groups, and in subcortical structures in RS + ELF-EMF group, this finding corroborates previous reports in which movement/synthesis of cholesterol is brain are area-dependent. Segatto, found a differential activity pattern of 3-hydroxy-3-methylglutaryl coenzyme A reductase in different brain regions with the highest activity in brain cortex and the lowest activity in brainstem [25]. Cholesterol is essential for membrane structure and stability, it decreases during stress and depressive-like behaviour in rats [26], and in neuronal diseases in human beings [27, 28]. Oliveira found that free cholesterol was the most abundant of the lipids in all brain regions analyzed, showing higher levels in cerebellum [29]. Results of the present work confirm the observation of highest cholesterol levels in cerebellum, and extend the finding of resistance to mild stressor conditions at this brain area.

Increased cholesterol levels found in cortex and subcortical structures in response to ELF-EMF exposure or RS + ELF-EMF could be an adaptive response for cellular protection, as observed in cerebellum. On the contrary, reduced cholesterol levels could affect the animal behaviour [26], suggesting a decrease in brain function. The increasing CT observed in the present study suggest that brain cortex CT turnover increases in response to ELF-EMF exposure, as seen in neuroinflammation and in other pathologic conditions, according to different reports [27, 28, 30]. On the other hand, we found a decrease in POL content of the cortex in the groups exposed to ELF-EMF, and RS + ELF-EMF groups, this finding is in accordance with previous reports in which some noxious conditions as maternal deprivation, RS, and ELF-EMF stimulation have effects on phospholipids and phospholipid-dependent pathways [20, 31].

According to our results, Oliveira [29] found POL changes in cortex and cerebellum after deep chronic stress, sphingolipid and phospholipid metabolism were deeply affected, showing a decrease in: phosphatidylethanolamine, ether phosphatidylcholine, and an increase in lysophosphatidylethanolamine levels similar to Lee et al. [32].

It has been observed that stress modifies the profile of mainly POL and fatty acid in different brain regions; in our results, we found a decrease of POL in cortex and in subcortical structures of ELF-EMF and RS + ELF-EMF groups, suggesting an association of this effect to ELF-EMF stimulation.

Fatty acids are usually bound to complex lipids in the cell membrane, and many stimulus can induce the breakdown of these complex lipids, which can be converted to signalling molecules, second messengers, and other molecules involved in neuronal metabolism and survival [33]. In the present study, we found an increase of NEFAs (as breakdown index for fatty acid released from complex lipids) in subcortical structures of rats exposed to RS + ELF-EMF, but not in other brain regions studied. We suggest that this increase in NEFAs could be through phospholipase activation by ELF-EMF, as described by Piacentini et al. [34]; nevertheless, the regional effects in NEFAs composition remains unclear [31, 35].

In further analysis, we found changes in total FAMEs composition in cortex, cerebellum and subcortical structures. According to our chromatographic method, eicosatetraenoic, docosahexaenoic, docosatetranoic, octadecanoic, and eicosenoic acids were found with major abundance in the tissues analyzed. Under physiologic conditions, the balance of membrane lipid metabolism, particularly of arachidonic and docosahexaenoic acids, lead a very small and tightly controlled cellular pool of free arachidonic acid, but their levels increase very quickly upon cell activation, cerebral ischemia, seizures or other types of brain stress [30, 35, 36]. However, in the present study a decrease in the relative abundance of FAMEs was found, especially in brain cortex in RS group. This effect could be due to membrane-bound fatty acids transformation into other metabolites as suggested by Malcher-Lopes et al. [24]; similar findings were observed in the cerebellum, with a decrease in eicosatetraenoic and docosahexaenoic acids in rats exposed to ELF-EMF, but not in the RS group, this may suggest that an ELF-EMF-mediated mechanism is involved in metabolism of these lipids, this mechanism could be through phospholipase activation as suggested by some authors [30, 37, 38]. We also observed a differential effect on the subcortical structures; RS induces a decrease in eicosatetraenoic and docosahexaenoic acids, while ELF-EMF stimulation induces an increase in these lipids. These findings agree with those reported by Clejan et al. [39], who found a differential effect of ELF-EMF on phospholipases and their patterns in second messengers in hematopoietic cell lines.

The cellular effects of extremely low frequency electromagnetic fields remain unknown, but several hypothesis about the mechanism of action have been proposed; one is the lifetime extension of free radicals, and radical-mediated damages on macromolecules [8, 40]. Some authors have hypothesized that ELF-EMF can act on living organisms in a similar way to other stressors, like heat and RS, by inducing the neuroendocrine stress response [41, 42]. According to that proposal, the findings that cortex and cerebellum of experimental groups showed higher lipoperoxide levels than control group, but without differences in subcortical structures, could be explained because these areas were the closest and most exposed to ELF-EMF, so producing free radicals that induce lipid damage and increase of saturated-/unsaturated-fatty acids ratio. In a previous report we observed that acute exposure to EMF induces reduction in catalase and superoxide dismutase activities, without changes in lipoperoxidation [11]. In the present study, lipid damage could be by induction of oxidative imbalance due to chronic exposure, suggesting that chronic ELF-EMF exposure could be like a mild-stressor; this finding is supported by the increase levels in plasma corticosterone concentration and brain lipid peroxidation. Changes in lipid composition, could have deep effects on membrane function by affecting membrane-associated enzymes, receptors and ion channels [43]. In the present study, different effects of RS and ELF-EMF were found in different brain regions, we speculate that these effects may be mediated by specific mechanisms, like phospholipase [30] activation by ELF-EMF and genomic and non-genomic effects of glucocorticoids, but these observations deserve further research as has been suggested previously in clinical trials [44].

## Conclusions

Changes found in the present study are in accordance with previous reports indicating the effects of chronic stress on brain lipid metabolism and suggest that the actions of extremely low frequency electromagnetic fields are similar to physiological stress. The effects found in different brain regions indicate that the extremely low frequency electromagnetic fields could be distance-dependent from the source of exposure due to findings in the analyzed brain regions. The effects in EMF versus RS were found differences on total cholesterol and polar lipids in cortex. In addition, in subcortical zone was found differences on total lipids. Although cerebellum’s lipid peroxidation induced by any stressor was similar to the found in cortex, minor changes in lipid profile were found in cerebellum and subcortical structures were more susceptible to increase NEFAs content in response to RS + ELF-EMF exposure.

## Abbreviations

ANOVA: analysis of variance
C: control
CNS: central nervous system
ELF-EMF: extremely low frequency electromagnetic field
ELISA: enzyme-linked immune sorbent assay
FAMEs: fatty acid methyl esters
GC: gas chromatography
HPA: hypothalamic–pituitary–adrenal
MDA: malondialdehyde
mT: milliTesla
NEFAs: non-esterified fatty acids
POL: polar lipids
RS: restraint stress
TAG: triacylglycerols
TBARS: thiobarbituric acid reactive substances
TC: total cholesterol
TL: total lipids
UNAM: Universidad Nacional Autónoma de México
